# Computational Screening of Bonding-Controlled Electronic Structures in One-Dimensional Cu/Ag-Based Hybrid Semiconductors

**DOI:** 10.3390/ma19071393

**Published:** 2026-03-31

**Authors:** Zhongwei Liu, Xiaoyu Yang, Xin He, Yuanhui Sun

**Affiliations:** 1 School of Materials Science and Engineering, Jilin University, Changchun 130012, China; liuzw25@mails.jlu.edu.cn (Z.L.); xiaoyuy22@mails.jlu.edu.cn (X.Y.); 2 Suzhou National Laboratory, Suzhou 215123, China

**Keywords:** hybrid organic–inorganic materials, first-principles calculation, Cu/Ag-based hybrid semiconductors

## Abstract

One-dimensional hybrid organic–inorganic semiconductors enable band-edge engineering through reduced dimensionality and interfacial orbital hybridization. Nevertheless, the electronic physics of Cu/Ag-based systems has received limited attention. Here, we perform high-throughput first-principles calculations on 90 Cu/Ag halide HOISs derived from experimentally reported parent structures to elucidate bonding-dependent electronic behavior. We uncover a clear transition from electronically isolated inorganic chains in ionic hybrids to strongly hybridized band edges in covalent and mixed-bonding hybrid frameworks, where ligand *p* orbitals cooperatively couple with Cu-derived states and halogen *p* orbitals. This hybridization produces *p*-orbital-dominated band edges, enhanced dispersion, and light-hole effective masses along the 1D chains. Guided by this bonding-driven mechanism, we further identify four Cu-based compounds, which are helpful for tuning light-harvesting properties in low-dimensional hybrid semiconductors.

## 1. Introduction

Hybrid organic–inorganic semiconductors (HOISs) integrate the structural diversity of organic components with the chemical robustness of inorganic frameworks, enabling highly tunable optoelectronic functionalities [1,2,3,4]. In particular, 1D HOISs exhibit pronounced structural anisotropy and reduced dimensionality, where quantum confinement and organic–inorganic interfacial interactions jointly modulate their electronic structures, optical responses, and charge transport [5]. As a result, 1D HOISs have emerged as promising candidates for photodetectors, light-emitting devices, and other optoelectronic applications [6,7,8].

The metal center plays a decisive role in defining the band-edge characteristics and emission mechanisms of 1D HOISs. Group-14 halides (Pb/Sn/Ge), constructed from corner-, edge-, or face-sharing MX_6_ octahedra, often display broadband emission originating from self-trapped excitons [9,10,11]. Sb/Bi-based halides are regarded as Pb-free alternatives with strong light harvesting, and Sb-based compounds can even achieve photoluminescence quantum yields approaching 100% [12,13]. Transition-metal hybrids (e.g., Mn, Fe) often show emission related to *d*-*d* transitions. Their photoluminescence lifetimes are typically long, ranging from microseconds to seconds [14]. In contrast, Cu/Ag-based HOISs feature closed-shell d^10^ electronic configurations, which enable ligand-assisted valence-band delocalization through metal–ligand coordination [15,16]. This distinct bonding motif provides an alternative route toward lightweight hole transport, fundamentally differing from the inorganic antibonding-dominated band edges in Pb/Sn systems [17,18,19]. Indeed, some Cu-based systems display extremely short fluorescence decay lifetimes, highlighting their potential for fast scintillators [20,21]. Ag-based hybrid materials have also demonstrated highly efficient broadband yellow emission [22]. Meanwhile, metal halide semiconductors based on Pb, Sb, and Bi have also shown significant promise in photovoltaic applications. In particular, Pb-based halide perovskites have achieved power conversion efficiencies exceeding 26% [23]. By comparison, Cu/Ag-based 1D HOISs remain far less explored despite their attractive band-structure features, tunable bonding environments, and suitable optoelectronic properties, particularly as light-absorbing materials for photovoltaic applications.

**Figure 1:**
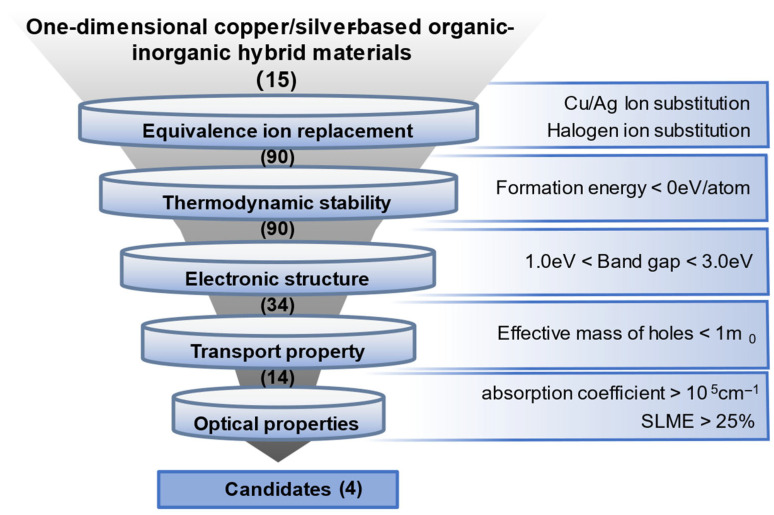
A multistep screening procedure employed to identify potential 1D Cu/Ag-based hybrid organic–inorganic materials. A total of 90 compounds were generated from 15 parent structures via equivalent ionic substitutions of Cu/Ag and halogen elements. The screening criteria were sequentially based on thermodynamic stability (formation energy < 0 eV/atom), electronic structure (1.0 eV < band gap < 3.0 eV), transport properties (effective mass < 1 m_0_), and optical properties (absorption coefficient > 10^5^ cm^−1^ and SLME > 25%).

Here, we propose a bonding-controlled paradigm for engineering band-edge states in 1D Cu/Ag hybrid semiconductors. By combining bonding-type classification with high-throughput density functional theory (DFT) screening, we reveal how organic–inorganic coordination fundamentally reshapes orbital hybridization, carrier dispersion, and optoelectronic response. Starting from 15 experimentally reported parent structures, we generate 90 compositions via isovalent substitutions at the Cu/Ag and Cl/Br/I sites. By sequentially evaluating thermodynamic stability, band gaps, carrier effective masses, and optical absorption/spectroscopic limited maximum efficiency (SLME) (Figure 1), we identify four outstanding candidates—(C_6_H_4_N_2_S)Cu_2_I_2_, (C_13_H_11_N)CuI, (C_6_H_4_N_2_Se)Cu_2_I_2_, and (C_5_H_5_O_2_N_3_)CuI—that simultaneously display suitable band gaps, strong near-edge absorption, and favorable hole transport. These results demonstrate the effectiveness of high-throughput screening in discovering lead-free 1D HOISs for photovoltaic applications and broader optoelectronic design.

## 2. Materials and Methods

High-throughput DFT calculations were performed using the plane-wave pseudopotential method as implemented in the Vienna ab initio Simulation Package(VASP 5.4.4) [24]. The DFT calculations were performed using a generalized gradient approximation exchange-correlation functional within the framework of the Perdew–Burke–Ernzerhof density functional [25]. The electron–ion interactions were described using the projector augmented-wave pseudopotentials [26]. During structural optimization, both lattice parameters and atomic positions were fully relaxed using the conjugate-gradient algorithm until the residual forces were smaller than 0.02 eV/Å. A plane-wave kinetic energy cutoff of 520 eV was adopted for the expansion of electronic wave functions. Brillouin zone integrations were carried out using a Monkhorst–Pack *k*-point mesh with a spacing of 2 π × 0.03 Å^−1^, while a denser mesh of 2 π × 0.01 Å^−1^ was employed for optical property calculations [27]. Long-range dispersion interactions between organic ligands and inorganic chains were accounted for using the DFT-D3 correction [28]. To remedy the band-gap underestimation of PBE, Heyd–Scuseria–Ernzerhof (HSE06) functional calculations were used to derive a scissors shift, which was subsequently applied to the PBE-based optical spectra. The SLME was evaluated to assess the optoelectronic potential of selected candidates [29]. The high-throughput first-principles calculations were conducted using the Jilin Artificial Intelligence-assisted Materials Design Integrated Platform (JAMIP), which is a data-driven open-source infrastructure for AI-assisted materials science research [30].

## 3. Results

### 3.1. Candidate Materials Preparation

**Figure 2:**
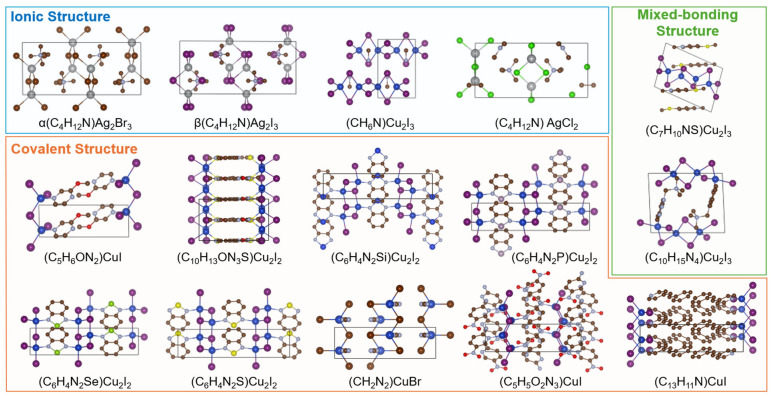
The blue boxed area represents ionic hybrid organic–inorganic materials, the orange boxed area denotes covalent hybrid materials, and the green boxed area corresponds to mixed-bonding hybrid materials.

We first screened 15 experimentally synthesized 1D HOISs without partial occupancy from the ICSD database [31], including four ionic-type, nine covalent-type, and two mixed-bonding hybrid type compounds (Figure 2). This classification is based on the nature of chemical bonding between the organic and inorganic components, which plays a crucial role in determining structural stability and electronic coupling [32]. Ionic hybrid halides (blue boxed area in Figure 2) consist of cationic organic ligands and anionic inorganic frameworks connected predominantly through electrostatic interactions without direct coordination bonds. Covalent hybrid materials (orange boxed area in Figure 2) are constructed via metal–ligand coordination, forming charge-neutral organic–inorganic frameworks. The third category (green boxed area in Figure 2) represents so-called mixed-bonding hybrid structures, in which ionic and coordination bonds coexist between organic and inorganic components.

Based on these parent structures, a total of 90 hybrid compounds were generated through systematic isovalent substitutions at the metal (Cu/Ag) and halide (Cl/Br/I) sites. Substitution of organic molecules was not considered in this work, as organic ligands show substantial configurational flexibility, and their replacement would likely alter the dimensionality and structural topology of the inorganic framework. A comprehensive investigation of organic–ligand effects will be addressed in future studies.

### 3.2. Stability Evaluation of Candidates

Considering that structure stability is critical for experimental synthesizability, we first evaluated the thermodynamic stability of the target hybrid compounds. The formation energy was calculated as follows [33]:

$$E_{form}=\frac{E_{HOIS}-nE_{inorg}^{ref}-mE_{L}}{N_{atom}}$$

Here, $E_{HOIS}$ and $E_{L}$ denote the total energies of HOISs and the organic molecule *L*, respectively. $E_{inorg}^{ref}$ represents the energy of the bulk inorganic semiconductor per formula unit. The parameter *n* is the number of inorganic chains, and *m* is the number of organic molecules. $N_{atom}$ is the total number of atoms. A formation energy of $E_{form}<$0 eV/atom indicates that the target compound is thermodynamically stable against decomposition into inorganic and organic constituents.

**Figure 3:**
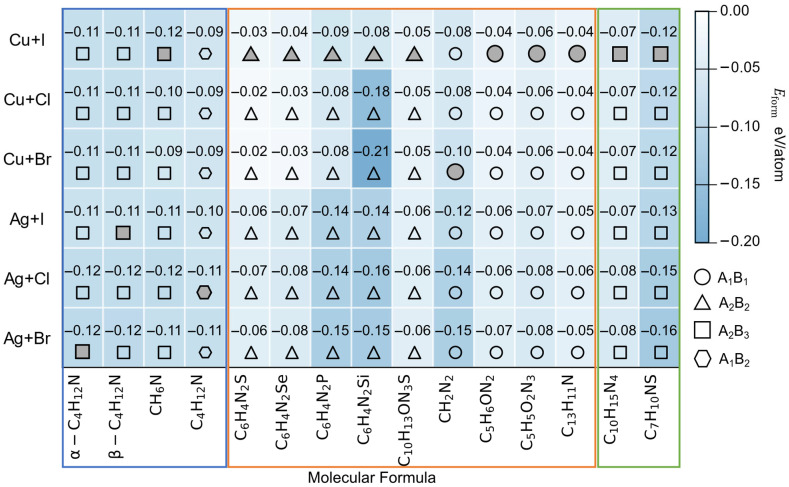
Ionic-, covalent-, and mixed-bonding hybrids are divided into blue, orange, and green regions, respectively. This classification is based on the nature of the chemical bonding between the organic and inorganic components. These structures are further classified into four types, A_1_B_1_, A_2_B_2_, A_2_B_3_, and A_1_B_2_, according to the ratio of their organic and inorganic components. A darker background color indicates a lower formation energy for the structure. Solid symbols indicate that the structure has been synthesized experimentally.

Thermodynamic calculations reveal a clear structure stability relationship across ionic-, covalent-, and mixed-bonding hybrid 1D Cu/Ag-based HOISs. Ionic hybrid halides (blue region) are the most stable owing to strong coulombic interactions, while covalent hybrid halides (orange region) and mixed-bonding hybrid halides (green region) show moderately reduced stability due to coordination-induced distortions and mixed bonding yet remain within an experimentally accessible formation-energy window. Certain specific structure types show consistently low stability (e.g., (C_13_H_11_N)CuI-prototype), irrespective of the choice of metal ions or halide anions. Although covalent hybrid halides are less stable than fully ionic or mixed-bonding hybrid structures, they still fall within an energetically accessible stability window. Notably, all materials marked with solid symbols in Figure 3 have been successfully synthesized experimentally, in good agreement with the theoretical predictions discussed above. This consistency further indicates that covalent structures can remain stable under appropriate conditions. In other words, different structural types exhibit distinct characteristics and advantages in terms of thermodynamic stability and synthetic feasibility, providing valuable guidance for the targeted design of materials in future studies.

### 3.3. Electronic Property Analysis

**Figure 4:**
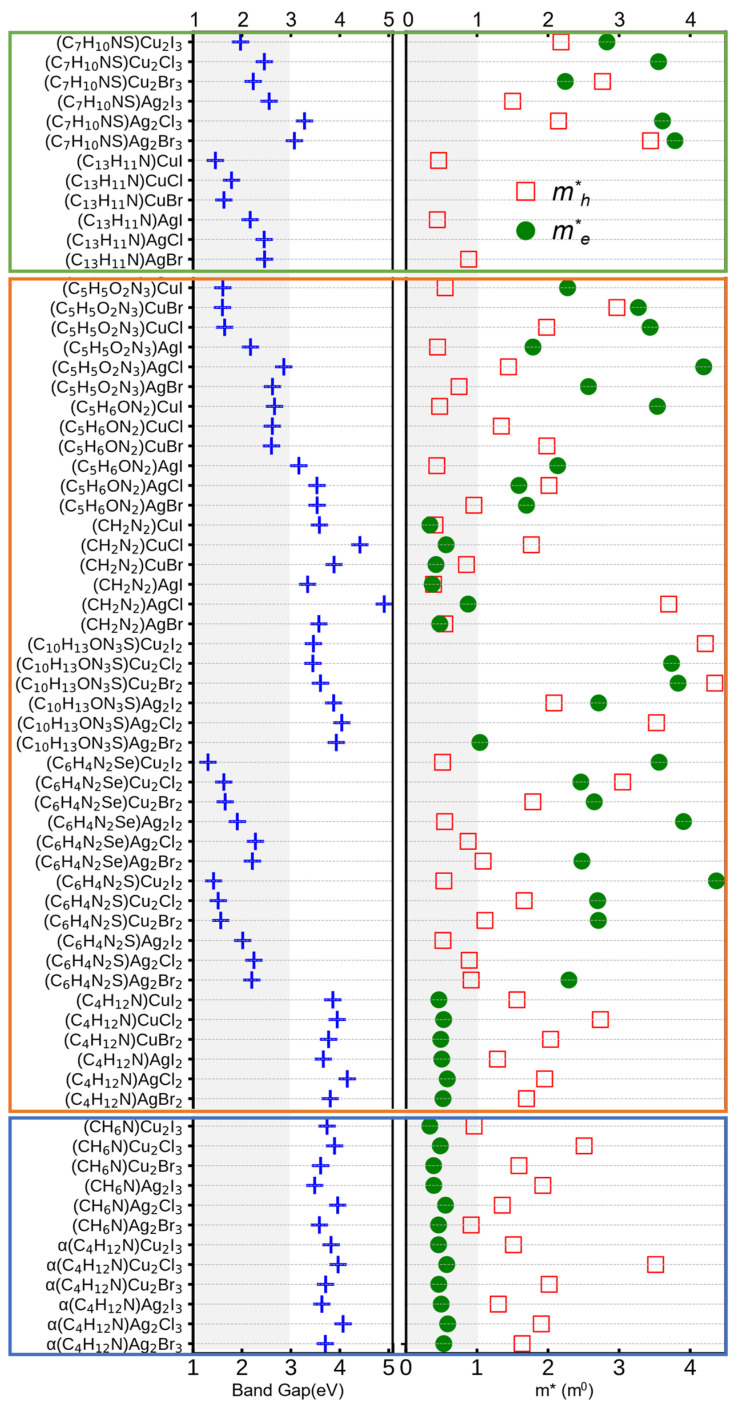
Ionic-, covalent-, and mixed-bonding hybrids are separated into blue, orange, and green regions, respectively. The **left** panel shows the band gap distribution, where ionic hybrid materials exhibit large band gaps. Although some covalent systems possess band gaps comparable to those of ionic compounds, a systematic reduction in band gap is mainly observed in mixed-bonding hybrid materials. The **right** panel presents the charge-carrier effective masses, which are extracted along the 1D inorganic chain direction. Green solid circles denote electron effective masses, while red open squares represent hole effective masses.

To obtain more accurate band-gap values, those calculated using the PBE functional were corrected by applying a scissors operator derived from HSE calculations for each compound, yielding more reliable estimations for the target compounds [33]. The corrected band gaps show good agreement with available experimental data, such as (C_6_H_4_N_2_S)Cu_2_I_2_ (calc: 1.58 eV, exp: 1.7 eV [34]), (C_5_H_6_ON_2_)CuI (calc: 2.65 eV, exp: 2.4 eV [35]). As shown in the left panel of Figure 4, ionic hybrid halides generally show wide band gaps exceeding 3.0 eV, arising primarily from the electronic decoupling between organic cations and inorganic frameworks. As shown in Figure S1, the valence band maximum (VBM) is mainly composed of Cu/Ag *d* and halogen *p* antibonding states, while the conduction band minimum (CBM) is dominated by metal *s*/*p* and halogen *p* orbitals. Meanwhile, the localized frontier orbitals of organic components lie energetically above the CBM, preventing effective orbital hybridization. Such materials typically consist of $[X_{m}Y_{m+n}{]}^{n-}$ (X = Cu, Ag; Y = Cl, Br, I) anionic frameworks electrostatically linked with organic cations, analogous to low-dimensional hybrid perovskites, and are therefore predominantly explored for self-trapped exciton-based luminescent applications [33,36,37,38]. In contrast, for covalent and mixed-bonding hybrid structures, the CBM exhibits significant contributions from organic molecular orbitals, indicating pronounced organic–inorganic hybridization. This coupling effectively narrows the band gaps and enhances optical absorption, rendering these materials more suitable for optoelectronic applications.

Carrier effective masses were further extracted from the electronic band structures. Considering the intrinsic anisotropy of 1D HOISs, electron and hole effective masses ($m_{e}^{*}$ and $m_{h}^{*}$) along the inorganic chain direction were adopted to characterize charge transport. Notably, two principal transport directions are present around the VBM and CBM. In this work, the effective masses $m_{e}^{*}$ and $m_{h}^{*}$ along the 1D chain direction were chosen to characterize charge-carrier transport, as these values more appropriately represent charge transport behavior and can better guide experimental investigations. As shown in the right panel of Figure 4, ionic hybrid halides typically show small electron effective masses (<1 m_0_) but relatively large hole effective masses, limiting their hole transport capability. Covalent hybrid materials display moderately distributed electron effective masses accompanied by substantially reduced hole effective masses. This behavior originates from metal–ligand coordination, which introduces organic-derived states near the VBM through structural distortions and orbital hybridization, leading to enhanced band dispersion and improved hole mobility. Mixed-bonding hybrid structures display smaller band gaps but more widely distributed effective masses, reflecting the sensitive dependence of their electronic structure on organic–inorganic coupling. However, carrier mobility is not determined solely by effective mass, but is also affected by scattering mechanisms (phonon scattering, defect scattering), possible polaron formation, and electron–phonon coupling, which often play important roles in hybrid materials. A more quantitative evaluation of carrier mobility would require explicit treatment of scattering and coupling effects.

Overall, these results indicate that covalent and mixed-bonding hybrids offer a favorable combination of suitable band gaps and low hole effective masses, highlighting their potential as hole-transporting semiconductors for optoelectronic applications. Based on these criteria (1.0–3.0 eV band gap and $m_{h}^{*}$ < 1 m_0_), fourteen candidate materials were identified for further optoelectronic screening.

### 3.4. Optoelectronic Property Screening

**Figure 5:**
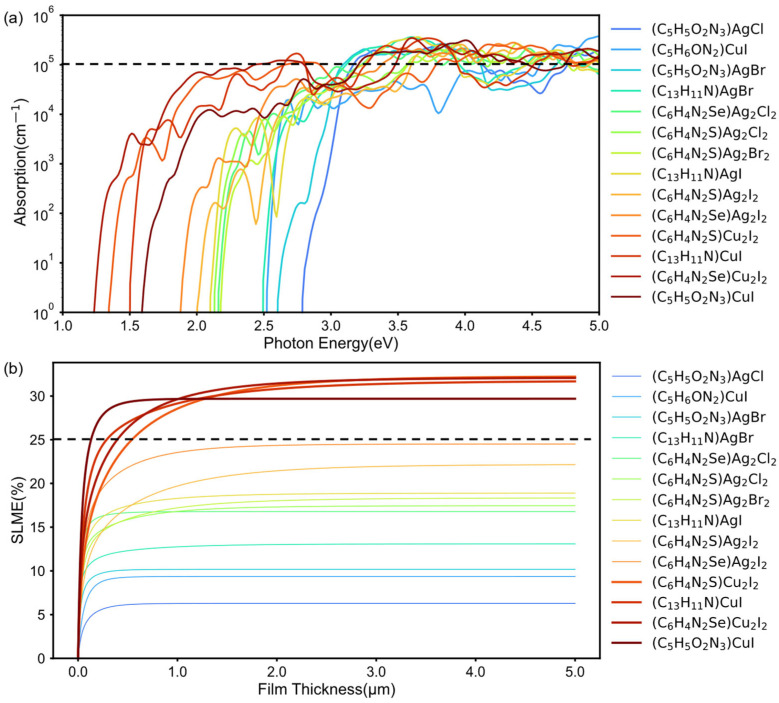
(**a**) Optical absorption spectra of hybrid materials satisfying the initial screening criteria. (**b**) SLME of the preliminarily screened materials, where (C_6_H_4_N_2_S)Cu_2_I_2_, (C_13_H_11_N)CuI, (C_6_H_4_N_2_Se)Cu_2_I_2_ and (C_5_H_5_O_2_N_3_)CuI all display SLME values exceeding 25%.

The optoelectronic properties of the candidate 1D HOISs were further screened with a focus on their potential application as hole-transporting layers. The initial selection criteria included negative formation energies ($E_{form}$ < 0 eV/atom), band gaps within the optimal solar spectrum window (1.0–3.0 eV), low hole effective masses ($m_{h}^{*}$ < 1.0 m_0_), and strong optical absorption (Figure 5a) near the band edge (>10^5^ cm^−1^). Based on these requirements, four promising materials—(C_6_H_4_N_2_S)Cu_2_I_2_, (C_13_H_11_N)CuI, (C_6_H_4_N_2_Se)Cu_2_I_2_, and (C_5_H_5_O_2_N_3_)CuI—were identified. The optoelectronic potential of these materials was further evaluated by calculating their SLME. This evaluation was based on a modified Shockley–Queisser (SQ) model [29], which incorporates the band gaps, the shape of the absorption spectrum near the threshold, and nonradiative recombination losses arising from material-dependent indirect–direct band gaps mismatches. As shown in Figure 5b, all four compounds show SLME values exceeding 25% at film thicknesses above 1 μm, which can be attributed to their favorable band gaps and strong absorption coefficients near the absorption edge.

**Figure 6:**
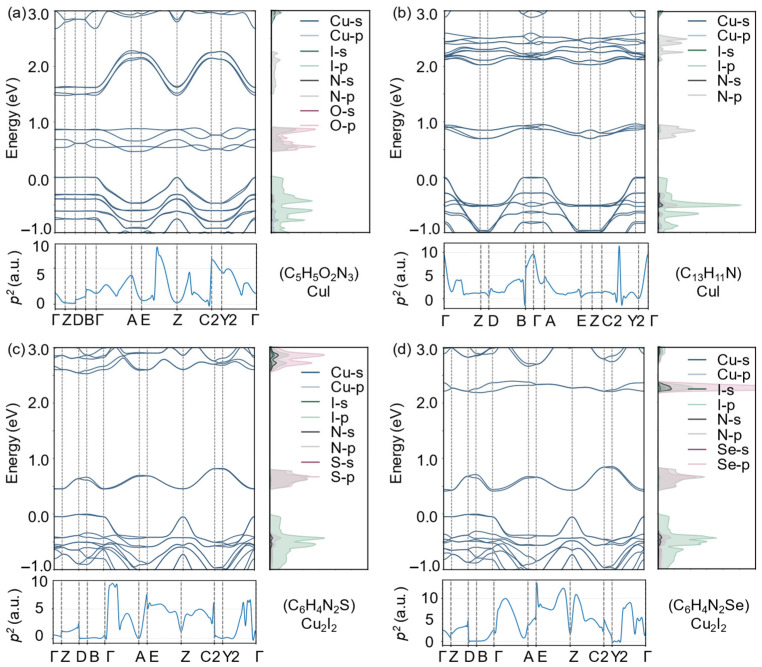
Each subpanel includes the band structure (left), PDOS (right), and $p^{2}$ (bottom). Panels (**a**–**d**) correspond to (C_6_H_4_N_2_S)Cu_2_I_2_, (C_13_H_11_N)CuI, (C_6_H_4_N_2_Se)Cu_2_I_2_, and (C_5_H_5_O_2_N_3_)CuI, respectively. The color coding in the band structures indicates the contributions of different elements and their orbitals (*s*/*p*), while the PDOS reflects the energy-resolved distribution of the corresponding orbitals. The $p^{2}$ indicator is used to characterize the variation in valence-to-conduction band optical transition probabilities across different *k* points.

To gain deeper insight into their electronic and optical characteristics, we analyzed the band structures, projected density of states (PDOS), and the square of transition dipole moment ($p^{2}$) of the four candidates (Figure 6a–d). HSE-corrected band gaps were adopted for quantitative discussion, while PBE-derived band dispersions and orbital projections were used to elucidate qualitative electronic trends [39,40]. The band-edge states are primarily composed of halogen *p* orbitals hybridized with Cu-derived states, together with significant contributions from N/O/S/Se *p* orbitals in the organic ligands, resulting in *p*-orbital-dominated valence and conduction band edges. Such orbital characteristics favor dipole-allowed interband transitions, leading to large optical transition matrix elements and enhanced absorption near the band edge. Consistently, pronounced $p^{2}$ peaks are observed around the band edges for all four systems, in agreement with their strong optical absorption and high SLME values.

From a charge-transport perspective, all four materials display noticeably larger band dispersion near the VBM compared to the CBM, indicating relatively lighter hole effective masses and preferential hole transport. The calculated effective masses along the 1D chain direction are: (C_6_H_4_N_2_S)Cu_2_I_2_ ($m_{e}^{*}$ = 0.53, $m_{h}^{*}$ = 0.44), (C_13_H_11_N)CuI ($m_{e}^{*}$ = 0.46, $m_{h}^{*}$ = 0.52), (C_6_H_4_N_2_Se)Cu_2_I_2_ ($m_{e}^{*}$ = 0.51, $m_{h}^{*}$ = 0.36), and (C_5_H_5_O_2_N_3_)CuI ($m_{e}^{*}$ = 0.55, $m_{h}^{*}$ = 0.27). Considering their suitable band gaps, favorable band-edge orbital hybridization, strong optical transitions, and efficient hole transport characteristics, all four systems emerge as promising candidates for optoelectronic applications, particularly as hole-transporting semiconductors.

Due to the one-dimensional crystal structure of these materials, their optical response exhibits pronounced polarization dependence. As shown in Figure S2, near the absorption edge, the absorption spectra along the chain direction (∥ chain) and perpendicular to the chain direction (⊥ chain) show significant differences. To quantitatively characterize this anisotropy, we define the absorption anisotropy ratio as

$$R(E)=\frac{\alpha_{∥}(E)}{\alpha_{⊥}(E)}$$

where $\alpha_{⊥}(E)$ represents the average absorption coefficient of the two orthogonal components perpendicular to the chain direction. Near the band edge, the maximum value of R(E) is significantly larger than 1, indicating that the optical transitions at the absorption edge exhibit strong polarization selectivity, with enhanced absorption along the chain direction. This polarization dependence originates from the anisotropic electronic structure associated with the Cu–I chains and the connected organic molecules. The band-edge states perpendicular to the chain direction mainly arise from the hybridization between the *p* orbitals of the inorganic chain and the *p* orbitals of N/O/S/Se atoms in the organic ligands, whereas the electronic states along the chain direction are primarily composed of orbitals within the inorganic framework (Cu-*p*/I-*p*).

### 3.5. Structure Property Correlations

**Figure 7:**
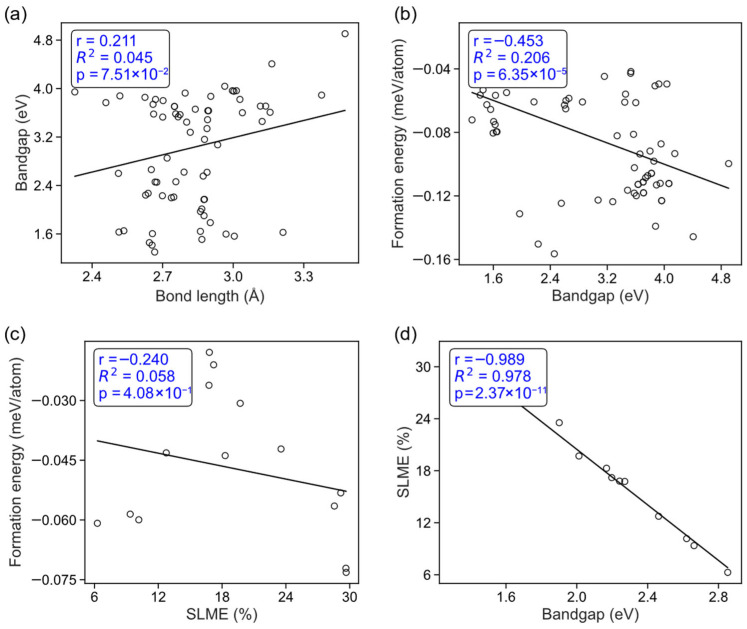
(**a**) Bandgap versus bond length shows a weak positive correlation. (**b**) Formation energy versus bandgap displays a moderate negative correlation. (**c**) Formation energy versus SLME exhibits a weak negative correlation. (**d**) SLME versus bandgap reveals a pronounced negative linear correlation. Open circles correspond to individual data points, while the solid black lines represent the linear regression fits. The inset in each panel summarizes the Pearson correlation coefficient (*r*), the coefficient of determination (*R*^2^), and the statistical significance (*p* value).

Based on the data points associated with bandgap and structural information, a systematic statistical analysis was performed to elucidate the relationships among bandgap, metal–halide bond length, stability, and SLME, as shown in Figure 7 and Figure S3. The overall dataset reveals only a weak positive correlation between bandgap and the average metal–halide bond length (Figure 7a, *r* = 0.21, *R*^2^ = 0.045, *p* = 0.075), indicating that simple geometric structural parameters have limited explanatory power for bandgap variation across the entire material family. Instead, the bandgap is still primarily governed by metal–halide orbital hybridization and the underlying electronic structure characteristics. In contrast, stability exhibits a moderately strong negative correlation with bandgap (Figure 7b, *r* = −0.45, *R*^2^ = 0.206, *p* = 6.35 × 10^−5^), suggesting that stronger metal–halide bonding generally corresponds to a lower bandgap while simultaneously leading to higher structural stability.

Analysis of the photovoltaic-performance-related descriptors further shows that there is almost no evident correlation between stability and SLME (Figure 7c, *R*^2^ = 0.058 and *p* = 0.41. This implies that, within this material system, improving photovoltaic efficiency does not necessarily come at the expense of structural stability, thereby offering the possibility of achieving both high stability and high efficiency in material design. In contrast, SLME shows an extremely strong negative correlation with bandgap (Figure 7d, *r* = −0.989, *R*^2^ = 0.978, *p* = 2.37 × 10^−11^), indicating that the spectroscopic limited maximum efficiency is predominantly controlled by the bandgap. This is consistent with the physical mechanism of the SLME model, in which the bandgap determines the position of the absorption edge and the efficiency of photogenerated carrier production.

Further classification of the system by metal species shows that both the Ag-based and Cu-based subsets preserve the same trend as the overall dataset, with stability remaining negatively correlated with bandgap (Figure S3, Ag: *R*^2^ = 0.336; Cu: *R*^2^ = 0.302). This suggests that metal substitution does not alter this fundamental structure–property relationship, although it does modulate the strength of the correlation to some extent. Similar behavior is also observed in the halide-substituted systems (Cl, Br, and I), where stability remains negatively correlated with bandgap in all three halide groups (Figure S3, *R*^2^ ≈ 0.16–0.24). This indicates that variation in halide species likewise does not change the basic coupling trend between stability and bandgap.

Overall, these statistical results demonstrate that the electronic structure and photovoltaic performance of this material system are primarily governed by the bandgap, which is in turn jointly influenced by the strength of metal–halide bonding and the chemical composition. A synergistic relationship exists between stability and bandgap, whereas no significant trade-off is found between stability and photovoltaic efficiency. These findings provide a clear materials-design strategy for simultaneously optimizing stability and photovoltaic performance through metal and halide substitution combined with local structural regulation.

## 4. Conclusions

In summary, we performed high-throughput first-principles calculations to systematically explore the thermodynamic stability, electronic structures, and optoelectronic properties of 90 1D Cu/Ag-based HOISs, aiming to expand the currently limited library of Cu/Ag 1D HOISs and to identify promising optoelectronic materials. Starting from 15 experimentally reported parent structures, 90 compounds were generated via isovalent substitutions at the metal (Cu/Ag) and halide (Cl/Br/I) sites, followed by a multistep screening workflow that sequentially evaluates formation energy, band gap, carrier effective mass, and optical absorption/SLME.

This bonding-dependent stability directly correlates with their electronic functionality—ionic hybrids typically exhibit wide band gaps (>3.0 eV) and large hole effective masses, making them less favorable for hole transport but suitable for luminescence-related applications, whereas covalent/mixed-bonding hybrid display pronounced band-edge organic–inorganic hybridization, yielding desirable band gaps in the 1.0–3.0 eV range and reduced hole effective masses along the 1D chain direction. By jointly screening stability, band-gap window, transport, and strong near-edge absorption (>10^5^ cm^−1^), we ultimately identify four standout candidates—(C_6_H_4_N_2_S)Cu_2_I_2_, (C_13_H_11_N)CuI, (C_6_H_4_N_2_Se)Cu_2_I_2_, and (C_5_H_5_O_2_N_3_)CuI—which feature p-orbital-dominated band edges, strong optical transition probabilities, efficient hole transport, and SLME values exceeding 25% for thicknesses >1 μm, thereby demonstrating that high-throughput first-principles screening is an effective route to expand the Cu/Ag-based 1D HOIS materials space and highlighting these Pb-free systems as a promising platform for light-absorbers in next-generation optoelectronic applications. This work establishes bonding engineering as an effective design principle for low-dimensional hybrid semiconductors.

## Data Availability

The original contributions presented in this study are included in the article/Supplementary Materials. Further inquiries can be directed to the corresponding authors.

## Supplementary Materials

The following supporting information can be downloaded at: https://www.mdpi.com/article/10.3390/ma19071393/s1, Figure S1: Electronic band structures and projected density of states (PDOS) of representative compounds for the three structural categories, namely (a) ionic hybrids, (b) covalent hybrids, and (c) mixed-bonding hybrids, in 1D Cu/Ag-based hybrid organic–inorganic semiconductors. For each structural category, Ag- and Cu-based compounds with Cl, Br, and I substitutions are presented. In each subpanel, the band structure is shown on the left and the corresponding PDOS on the right. The orbital-resolved PDOS reveals the respective contributions of metal, halogen, and ligand atoms to the band-edge states, illustrating how bonding type and chemical substitution influence orbital hybridization and electronic structure; Figure S2: Polarization-dependent absorption spectra of four representative Cu-based 1D hybrid organic–inorganic semiconductors. (a) (C_6_H_4_N_2_S)Cu_2_I_2_, (b) (C_6_H_4_N_2_Se)Cu_2_I_2_, (c) (C_5_H_5_O_2_N_3_)CuI, and (d) (C_13_H_11_N)CuI. In each panel, the upper plot shows the calculated absorption coefficients along the three crystallographic directions (*x*, *y*, and *z*), while the lower plot presents the absorption anisotropy ratio, defined as $\alpha_{∥}/\alpha_{⊥}$. The results reveal pronounced polarization dependence near the absorption edge, indicating strong optical anisotropy in these one-dimensional hybrid systems. The enhanced absorption along the chain direction originates from the anisotropic electronic structure associated with the inorganic Cu–I chains and their coupling with the organic ligands; Figure S3: Structure–property correlation analysis categorized by halide substitution (Cl, Br, and I) and metal substitution (Ag and Cu). The first row shows the relationship between bandgap and average bond length, the second row shows SLME versus bandgap, the third row shows formation energy versus bandgap, and the fourth row shows formation energy versus SLME. The results indicate that SLME consistently exhibits a strong negative correlation with bandgap across different chemical subsets, while formation energy generally shows a negative correlation with bandgap, suggesting an intrinsic coupling among bandgap, stability, and photovoltaic performance. In contrast, the correlations between bandgap and average bond length, as well as between formation energy and SLME, are comparatively weak. The solid black lines represent linear fitting results, and the inset in each panel lists the Pearson correlation coefficient (r), coefficient of determination (*R*^2^), and *p* value.

## References

[1] Xue J., Wang R., Chen X., Yao C., Jin X., Wang K.-L., Huang W., Huang T., Zhao Y., Zhai Y. (2021). Reconfiguring the Band-Edge States of Photovoltaic Perovskites by Conjugated Organic Cations. Science.

[2] Guo S., Li Y., Mao Y., Tao W., Bu K., Fu T., Zhao C., Luo H., Hu Q., Zhu H. (2022). Reconfiguring Band-Edge States and Charge Distribution of Organic Semiconductor–Incorporated 2D Perovskites via Pressure Gating. Sci. Adv..

[3] Fan X., Zhang M., Wang X., Yang F., Meng X. (2013). Recent Progress in Organic–Inorganic Hybrid Solar Cells. J. Mater. Chem. A.

[4] Zhou K., Qi B., Liu Z., Wang X., Sun Y., Zhang L. (2024). Advanced Organic–Inorganic Hybrid Materials for Optoelectronic Applications. Adv. Funct. Mater..

[5] Li Y., Hu Z., Guo Q., Li J., Liu S., Xie X., Zhang X., Kang L., Li Q. (2025). Van Der Waals One-Dimensional Atomic Crystal Heterostructure Derived from Carbon Nanotubes. Chem. Soc. Rev..

[6] Ott C., Reiter F., Baumgartner M., Pielmeier M., Vogel A., Walke P., Burger S., Ehrenreich M., Kieslich G., Daisenberger D. (2019). Flexible and Ultrasoft Inorganic 1D Semiconductor and Heterostructure Systems Based on SnIP. Adv. Funct. Mater..

[7] Zhao X., Du Y., Li W., Zhao Z., Lei M. (2023). Organic/Inorganic Hybrids for Intelligent Sensing and Wearable Clean Energy Applications. Adv. Compos. Hybrid Mater..

[8] Sakurada T., Cho Y., Paritmongkol W., Lee W.S., Wan R., Su A., Shcherbakov-Wu W., Müller P., Kulik H.J., Tisdale W.A. (2023). 1D Hybrid Semiconductor Silver 2,6-Difluorophenylselenolate. J. Am. Chem. Soc..

[9] Xu L.-J., Lin X., He Q., Worku M., Ma B. (2020). Highly Efficient Eco-Friendly X-Ray Scintillators Based on an Organic Manganese Halide. Nat. Commun..

[10] Kopacic I., Friesenbichler B., Hoefler S.F., Kunert B., Plank H., Rath T., Trimmel G. (2018). Enhanced Performance of Germanium Halide Perovskite Solar Cells through Compositional Engineering. ACS Appl. Energy Mater..

[11] Wang S., Mitzi D.B., Feild C.A., Guloy A. (1995). Synthesis and Characterization of [NH2C(I):NH2]3MI5 (M = Sn, Pb): Stereochemical Activity in Divalent Tin and Lead Halides Containing Single. Ltbbrac.110.Rtbbrac. Perovskite Sheets. J. Am. Chem. Soc..

[12] Noculak A., Morad V., McCall K.M., Yakunin S., Shynkarenko Y., Wörle M., Kovalenko M.V. (2020). Bright Blue and Green Luminescence of Sb(III) in Double Perovskite Cs_2_MInCl_6_ (M = Na, K) Matrices. Chem. Mater..

[13] Zhou C., Lin H., Tian Y., Yuan Z., Clark R., Chen B., van de Burgt L.J., Wang J.C., Zhou Y., Hanson K. (2017). Luminescent Zero-Dimensional Organic Metal Halide Hybrids with near-Unity Quantum Efficiency. Chem. Sci..

[14] Tao P., Liu S.-J., Wong W.-Y. (2020). Phosphorescent Manganese(II) Complexes and Their Emerging Applications. Adv. Opt. Mater..

[15] Zhou B., Yan D. (2023). Long Persistent Luminescence from Metal–Organic Compounds: State of the Art. Adv. Funct. Mater..

[16] Meng X.-D., Li X.-Y., Yang Y., Xue Z.-Z., Pan J. (2020). Construction of a Transition Metal Complex Directed Iodocuprate as the Visible Light Driven Photocatalyst. Inorg. Chem. Commun..

[17] Arora N., Dar M.I., Hinderhofer A., Pellet N., Schreiber F., Zakeeruddin S.M., Grätzel M. (2017). Perovskite Solar Cells with CuSCN Hole Extraction Layers Yield Stabilized Efficiencies Greater than 20%. Science.

[18] Zhao K., Munir R., Yan B., Yang Y., Kim T., Amassian A. (2015). Solution-Processed Inorganic Copper(i) Thiocyanate (CuSCN) Hole Transporting Layers for Efficient p–i–n Perovskite Solar Cells. J. Mater. Chem. A.

[19] Etgar L. (2015). Hole-Transport Material-Free Perovskite-Based Solar Cells. MRS Bull..

[20] Perruchas S., Le Goff X.F., Maron S., Maurin I., Guillen F., Garcia A., Gacoin T., Boilot J.-P. (2010). Mechanochromic and Thermochromic Luminescence of a Copper Iodide Cluster. J. Am. Chem. Soc..

[21] Liu Z., Qiu J., Wei F., Wang J., Liu X., Helander M.G., Rodney S., Wang Z., Bian Z., Lu Z. (2014). Simple and High Efficiency Phosphorescence Organic Light-Emitting Diodes with Codeposited Copper(I) Emitter. Chem. Mater..

[22] Yang H., Mandal S., Lee Y.H., Park J.Y., Zhao H., Yuan C., Huang L., Chen M., Dou L. (2023). Dimensionality Engineering of Lead Organic Chalcogenide Semiconductors. J. Am. Chem. Soc..

[23] Liang L., Nan Z.-A., Li Y., Zhang Y., Fei Z., Shibayama N., Zhang Z., Lin Z., Chen W., Li C. (2025). Formation Dynamics of Thermally Stable 1D/3D Perovskite Interfaces for High-Performance Photovoltaics. Adv. Mater..

[24] Kresse G., Furthmüller J. (1996). Efficient Iterative Schemes for Ab Initio Total-Energy Calculations Using a Plane-Wave Basis Set. Phys. Rev. B.

[25] Perdew J.P., Burke K., Ernzerhof M. (1996). Generalized Gradient Approximation Made Simple. Phys. Rev. Lett..

[26] Blöchl P.E. (1994). Projector Augmented-Wave Method. Phys. Rev. B.

[27] Monkhorst H.J., Pack J.D. (1976). Special Points for Brillouin-Zone Integrations. Phys. Rev. B.

[28] Schröder H., Hühnert J., Schwabe T. (2017). Evaluation of DFT-D3 Dispersion Corrections for Various Structural Benchmark Sets. J. Chem. Phys..

[29] Yu L., Zunger A. (2012). Identification of Potential Photovoltaic Absorbers Based on First-Principles Spectroscopic Screening of Materials. Phys. Rev. Lett..

[30] Zhao X.-G., Zhou K., Xing B., Zhao R., Luo S., Li T., Sun Y., Na G., Xie J., Yang X. (2021). JAMIP: An Artificial-Intelligence Aided Data-Driven Infrastructure for Computational Materials Informatics. Sci. Bull..

[31] Bergerhoff G., Hundt R., Sievers R., Brown I.D. (1983). The Inorganic Crystal Structure Data Base. J. Chem. Inf. Comput. Sci..

[32] Liu W., Fang Y., Li J. (2018). Copper Iodide Based Hybrid Phosphors for Energy-Efficient General Lighting Technologies. Adv. Funct. Mater..

[33] Li Y., Yang J., Zhao R., Zhang Y., Wang X., He X., Fu Y., Zhang L. (2022). Design of Organic–Inorganic Hybrid Heterostructured Semiconductors via High-Throughput Materials Screening for Optoelectronic Applications. J. Am. Chem. Soc..

[34] Fang Y., Zhu K., Teat S.J., Reid O.G., Hei X., Zhu K., Fang X., Li M., Sojdak C.A., Cotlet M. (2022). Robust and Highly Conductive Water-Stable Copper Iodide-Based Hybrid Single Crystals. Chem. Mater..

[35] Xu C., Lv L., Luo D., Liu W. (2020). Synthesis, Structure and Photoluminescence Properties of Three Copper(i) Iodide Based Inorganic–Organic Hybrid Structures with Pyrazine Derivatives. New J. Chem..

[36] Xu T., Li Y., Nikl M., Kucerkova R., Zhou Z., Chen J., Sun Y.-Y., Niu G., Tang J., Wang Q. (2022). Lead-Free Zero-Dimensional Organic-Copper(I) Halides as Stable and Sensitive X-Ray Scintillators. ACS Appl. Mater. Interfaces.

[37] Mao P., Tang Y., Wang B., Fan D., Wang Y. (2022). Organic–Inorganic Hybrid Cuprous Halide Scintillators for Flexible X-Ray Imaging. ACS Appl. Mater. Interfaces.

[38] Lian L., Zhang T., Ding H., Zhang P., Zhang X., Zhao Y.-B., Gao J., Zhang D., Zhao Y.S., Zhang J. (2022). Highly Luminescent Zero-Dimensional Organic Copper Halide with Low-Loss Optical Waveguides and Highly Polarized Emission. ACS Mater. Lett..

[39] Kim B., Kim J., Park N. (2020). First-Principles Identification of the Charge-Shifting Mechanism and Ferroelectricity in Hybrid Halide Perovskites. Sci. Rep..

[40] Borlido P., Schmidt J., Huran A.W., Tran F., Marques M.A.L., Botti S. (2020). Exchange-Correlation Functionals for Band Gaps of Solids: Benchmark, Reparametrization and Machine Learning. npj Comput. Mater..

