# Supplementary material for: Computational Screening of Bonding-Controlled Electronic Structures in One-Dimensional Cu/Ag-Based Hybrid Semiconductors
Source: Materials (Basel). 2026 Mar 31;19(7):1393. doi: 10.3390/ma19071393 (PMC13074672; doi:10.3390/ma19071393)
Supplement: Supplementary file 1 [file materials-19-01393-s001.zip › materials-4163956-supplementary.pdf]

Supporting Information

Computational screening of bonding-controlled electronic structures in one-dimensional Cu/Ag-based hybrid semicon-ductors

Zhongwei Liu[1, 2], *Xiaoyu Yang*[1], *Xin He*[1,*], *Yuanhui Sun*[2,*]

[1] School of Materials Science and Engineering, Jilin University, Changchun 130012, China

[2] Suzhou National Laboratory, Suzhou, 215123, China

* Correspondence: xin_he@jlu.edu.cn; sunyh@szlab.ac.cn

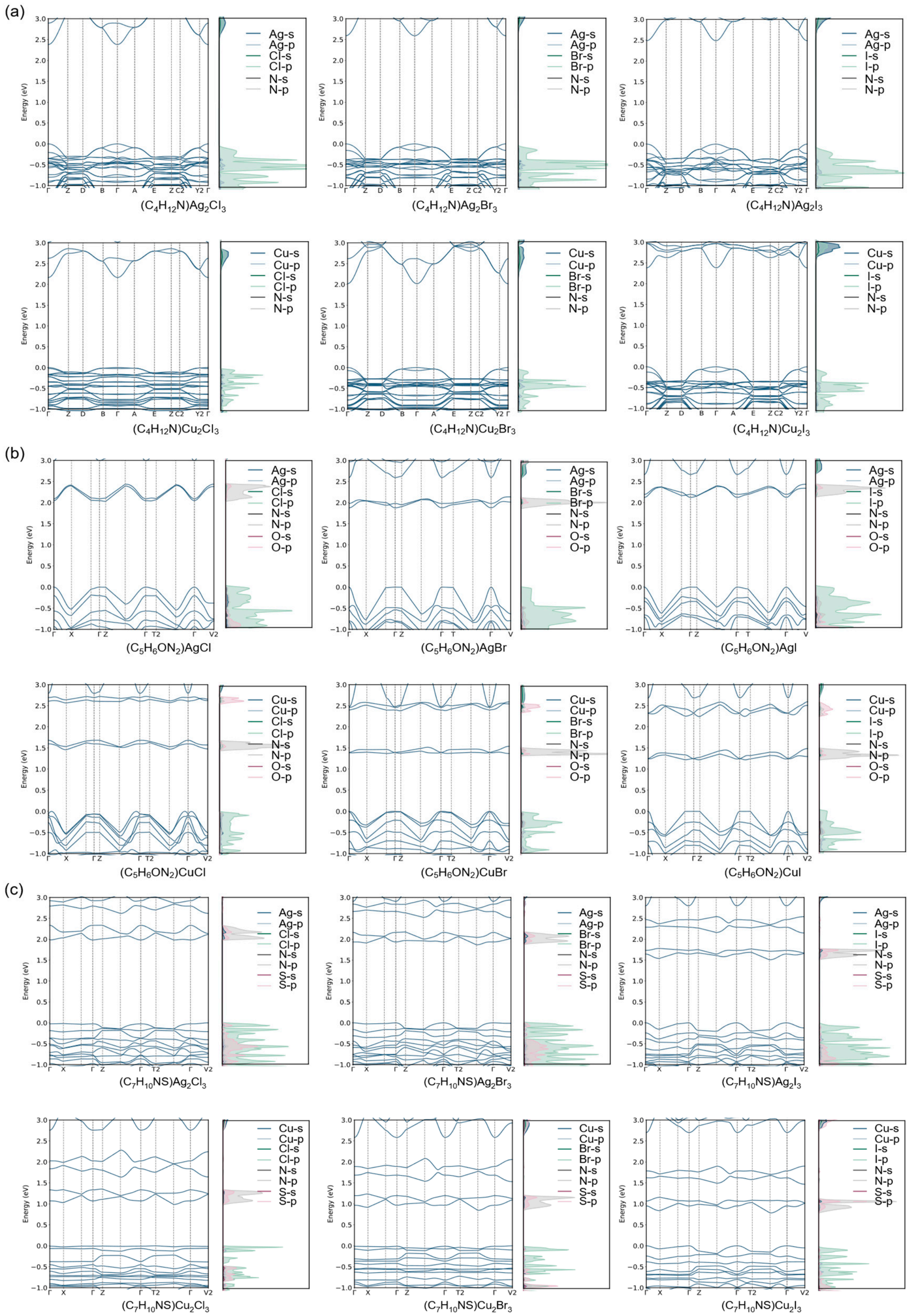

Figure S1. Electronic band structures and projected density of states (PDOS) of representative compounds for the three structural categories, namely (a) ionic hybrids, (b) covalent hybrids, and (c) mixed-bonding hybrids, in 1D Cu/Ag-based hybrid organic–inorganic semiconductors. For each structural category, Ag- and Cu-based compounds with Cl, Br, and I substitutions are presented. In each subpanel, the band structure is shown on the left and the corresponding

PDOS on the right. The orbital-resolved PDOS reveals the respective contributions of metal, halogen, and ligand atoms to the band-edge states, illustrating how bonding type and chemical substitution influence orbital hybridization and electronic structure.

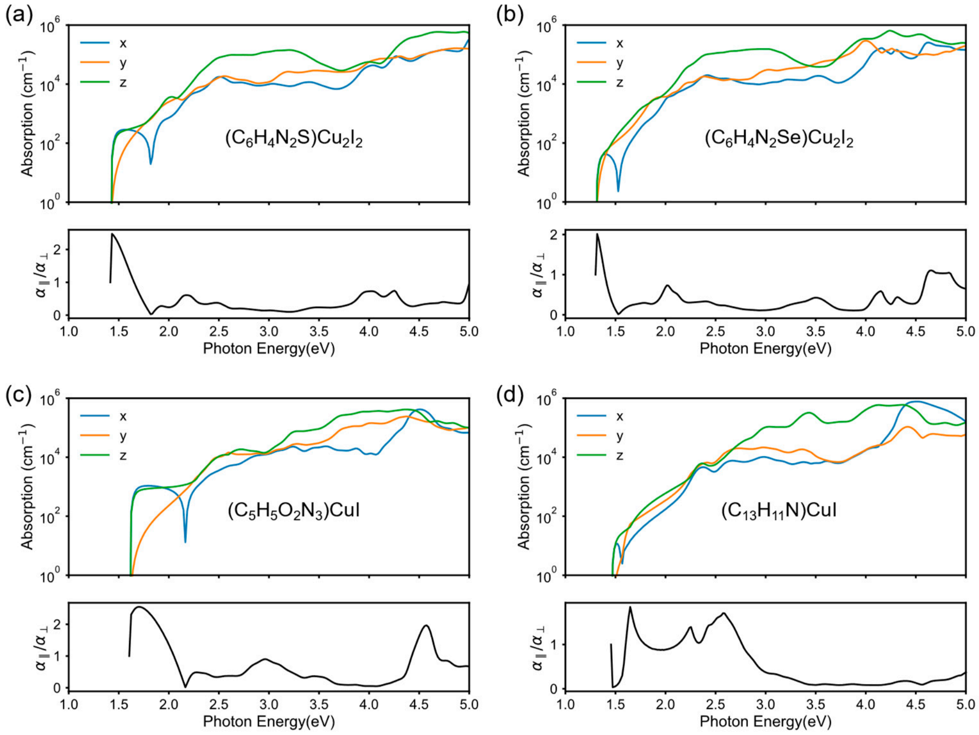

Figure S2. Polarization-dependent absorption spectra of four representative Cu-based 1D hybrid organic–inorganic semiconductors. (a) $(C_6H_4N_2S)Cu_2I_2$, (b) $(C_6H_4N_2Se)Cu_2I_2$, (c) $(C_5H_5O_2N_3)CuI$, and (d) $(C_{13}H_{11}N)CuI$. In each panel, the upper plot shows the calculated absorption coefficients along the three crystallographic directions (x, y, and z), while the lower plot presents the absorption anisotropy ratio, defined as $\alpha_{\parallel}/\alpha_{\perp}$. The results reveal pronounced polarization dependence near the absorption edge, indicating strong optical anisotropy in these one-dimensional hybrid systems. The enhanced absorption along the chain direction originates from the anisotropic electronic structure associated with the inorganic Cu–I chains and their coupling with the organic ligands.

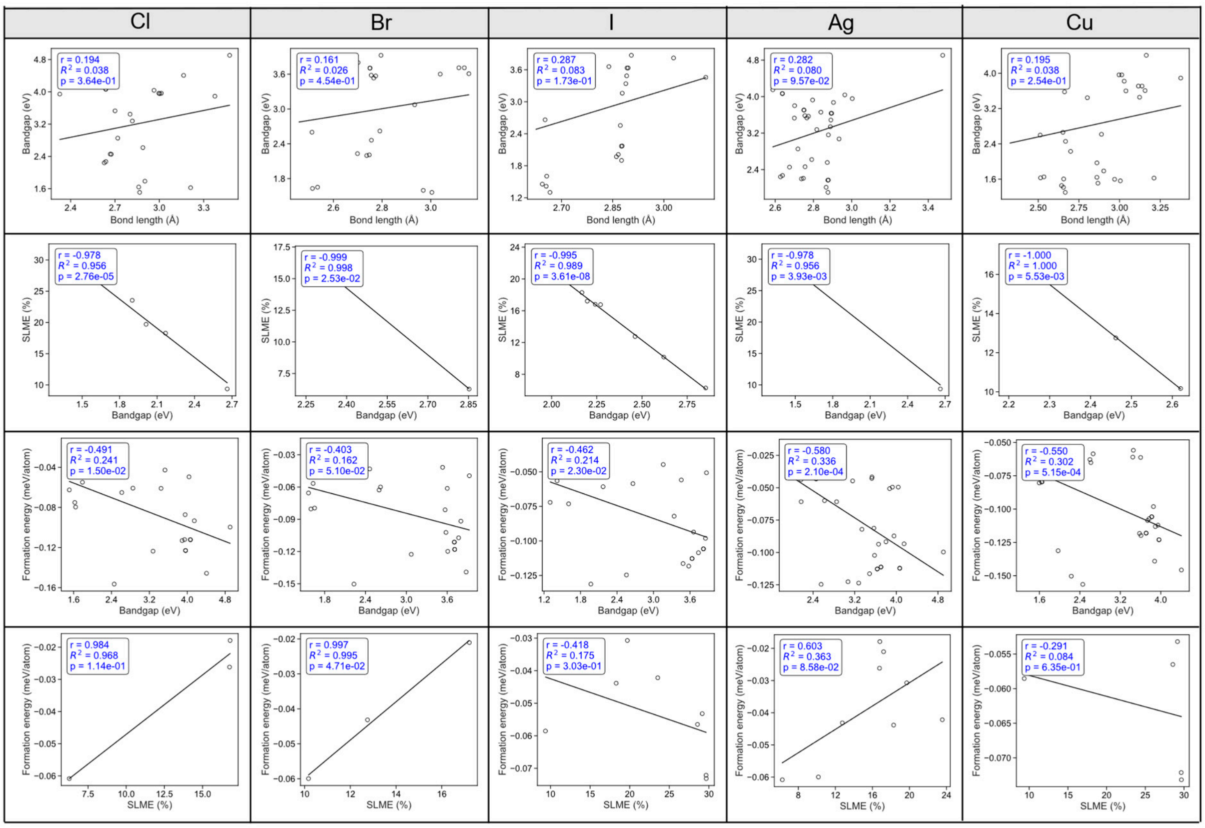

Figure S3. Structure-property correlation analysis categorized by halide substitution (Cl, Br, and I) and metal substitution (Ag and Cu). The first row shows the relationship between bandgap and average bond length, the second row shows SLME versus bandgap, the third row shows formation energy versus bandgap, and the fourth row shows formation energy versus SLME. The results indicate that SLME consistently exhibits a strong negative correlation with bandgap across different chemical subsets, while formation energy generally shows a negative correlation with bandgap, suggesting an intrinsic coupling among bandgap, stability, and photovoltaic performance. In contrast, the correlations between bandgap and average bond length, as well as between formation energy and SLME, are comparatively weak. The solid black lines represent linear fitting results, and the inset in each panel lists the Pearson correlation coefficient (r), coefficient of determination (R^2), and p value.
